# Evaluating the role of lipopolysaccharides in the joint: fibronectin as a novel protective mechanism

**DOI:** 10.1136/rmdopen-2025-005622

**Published:** 2025-07-10

**Authors:** Kajetana Bevc, Shipin Zhang, Andres Pazos-Perez, Ana Alonso-Perez, David Fercher, Sami Kauppinen, Tuomas Frondelius, Valentino Bruhin, Gian Salzmann, Thomas Rauer, Hans-Christoph Pape, Mikko Arttu Jalmari Finnilä, Caroline Ospelt, Rodolfo Gomez, Kari K Eklund, Marcy Zenobi Wong, Goncalo Barreto

**Affiliations:** 1 D-HEST, ETH Zurich, Zürich, Switzerland; 2 University Hospital of Santiago de Compostela, Santiago de Compostela, Spain; 3 Research Group of Medical Imaging, Physics and Technology, University of Oulu, Oulu, Finland; 4 Schulthess Klinik, Zürich, Switzerland; 5 Department of Traumatology, University Hospital Zurich, Zürich, Switzerland; 6 Center of Experimental Rheumatology, University Hospital Zurich, Zürich, Switzerland; 7 Department of Rheumatology, Helsinki University Central Hospital, Helsinki, Finland; 8 Clinicum, University of Helsinki Faculty of Medicine, Helsinki, Finland

**Keywords:** Synovial fluid, Inflammation, Osteoarthritis, Knee

## Abstract

**Objective:**

To investigate the presence and bioactivity of lipopolysaccharides (LPS) in synovial fluid (SF) of osteoarthritis (OA) patients and elucidate mechanisms modulating their inflammatory potential.

**Methods:**

SF samples from 56 OA, 7 rheumatoid arthritis and 39 trauma patients were analysed for LPS concentration and bioactivity. Lipid A composition was assessed using liquid chromatography–mass spectrometry (LC-MS). In a rat model, LPS was administered systemically for 32 days to evaluate its impact on joint degeneration. The interaction between LPS and synovial proteins, particularly fibronectin (Fn), was examined through in vitro assays and a 3D synovial membrane model.

**Results:**

LPS was detected in all SF samples with comparable concentrations and lipid A profiles across all groups. LC-MS measurements indicated higher LPS levels than those obtained from standard endotoxin assays, suggesting limitations in conventional detection methods. Despite elevated LPS presence, bioactivity assays revealed minimal proinflammatory responses, implying the existence of intrinsic SF factors neutralising LPS. In vivo, prolonged systemic LPS exposure did not induce OA-like changes in rat joints. Notably, LPS colocalised with Fn in the synovial membrane, and Fn binding attenuated LPS bioactivity and hindered its migration in vitro.

**Conclusions:**

LPS is prevalent in SF across various joint conditions but exhibits low bioactivity, indicating it is not a primary driver of joint inflammation. Fn plays a crucial role in sequestering and neutralising LPS within the synovial environment, offering a protective mechanism against LPS-induced inflammation. These findings underscore the need for accurate LPS measurement techniques and suggest potential therapeutic targets for modulating joint inflammation.

**WHAT IS ALREADY KNOWN ON THIS TOPIC**

- Elevated serum lipopolysaccharide (LPS) levels are linked to joint inflammation and severity in osteoarthritis (OA) and rheumatoid arthritis (RA), likely due to increased gut permeability. LPS has been found in synovial fluid, but its activity and regulation by local factors remain unclear.

**WHAT THIS STUDY ADDS**

- LPS is present in synovial fluid from OA, RA and trauma patients at similar levels but exerts low proinflammatory activity. Fibronectin binds and neutralises LPS, revealing a protective synovial mechanism against LPS-induced inflammation.

**HOW THIS STUDY MIGHT AFFECT RESEARCH, PRACTICE OR POLICY**

- This study challenges the view of intra-articular LPS as a direct driver of joint inflammation and highlights fibronectin’s protective role, hopefully inspiring avenues for novel therapeutic strategies to mitigate inflammation in joint diseases.

## Introduction

 Lipopolysaccharide (LPS), also known as endotoxin, is a component of the outer cell wall of Gram-negative bacteria. It is composed of the conserved lipid A anchoring LPS to the cell wall and a structurally variable polysaccharide surface layer.^1^ LPS differs between different bacterial species and strains. These variations in LPS structure have an effect on the inflammatory response of the host and can be used to identify bacterial species from which LPS has been shed from.^2^ Lipid A is the primary immunostimulatory region of LPS that strongly activates toll-like receptor 4 (TLR4).^3^ When activated, it triggers translocation of nuclear factor ‘kappa-light-chain enhancer’ of activated B cells (NF-κB) to the nucleus, which results in an innate immune signalling cascade.^4^ NF-κB signalling is involved in arthritis pathology.^5^ LPS can translocate from the intestine into the bloodstream^6 7^ when intestinal porosity is increased.^8 9^

Blood LPS levels have been reported to correlate with arthritis.^10^,^12^ Osteoarthritis (OA) and rheumatoid arthritis (RA) are distinct yet debilitating joint diseases. OA is a chronic multitissue disease driven by an interplay of inflammation and mechanical damage, while RA is a systemic autoimmune disorder.^13^ Both diseases substantially decrease the patients’ quality of life.^13^,^15^ Loeser *et al* analysed serum samples of OA patients and healthy controls, finding LPS levels elevated in OA serum, however, they observed no correlation between LPS concentration and joint pain.^16^ In contrast, some papers suggest that LPS concentration in the serum is associated with radiographic knee joint space narrowing,^11^ knee osteophyte severity and abundance of macrophages in the synovium.^12^ Literature suggests that increased intestinal permeability^17^ in OA patients increases plasma LPS concentration,^12^ which can correlate with OA pathology.^11 12^ Similarly, serum LPS bioactivity has been linked with disease activity and likelihood of remission in RA patients.^10^

Little is known about LPS in synovial fluid (SF), which is important for OA pathogenesis.^18 19^ It has been reported that LPS is present in the SF of OA patients and is associated with knee osteophyte severity and the total WOMAC score.^12^ Kasperkiewicz *et al* also reported that anti-LPS antibodies were found in the SF of juvenile idiopathic arthritis patients.^20^ However, to the best of our knowledge, no report has been published comparing LPS concentration in SF to any control group nor investigating its biological activity. The presence of LPS in the serum and SF as well as its correlation to pathology of arthritis suggest it plays a role in the inflammation of the joint. However, the direct effect of endotoxemia as well as intra-articular LPS on joint inflammation or degeneration is largely unknown.

Because LPS is a potent activator of the immune system and a ubiquitous molecule, there are enzymes and proteins present in the serum and liver to degrade and reduce the biological activity of LPS.^21^ Resistin as well as high concentrations of LPS binding protein and soluble TLRs have been described to block LPS-mediated inflammation in the serum.^22^,^24^ We hypothesise that similar protective mechanisms could be found also in the SF, as SF is an ultrafiltrate of plasma^18^ and because soluble TLRs have been found elevated in OA SF.^25^ Another component of SF with an influence on bacterial adherence and virulence is fibronectin (Fn).^26^,^28^ Fn is present in healthy SF and elevated in OA SF.^26 29^ In the context of arthritis citrullinated Fn and Fn’s fragments have been identified as proinflammatory,^30^,^33^ however, its role in interacting with LPS in the joint has not yet been explored.

We hypothesised that LPS can contribute to joint inflammation, however that its bioactivity may be regulated by factors in the SF. To put the study to a clinical context, we quantified and compared LPS levels and bioactivity in SF from end-stage knee OA patients and traumatic knee injury patients. We also tested the effect of induced endotoxaemia on knee joint cartilage degradation, osteophyte formation and synovitis in vivo. We further searched for potential protective mechanisms in SF against effects of LPS.

## Methods

### Study participants

A total of 103 participants aged 18–90 were recruited based on the following inclusion criteria: for OA patients (male and female) undergoing a total knee arthroplasty, for RA patients (male and female) diagnosis of RA, for the trauma cohort no diagnosis of OA or RA, but a traumatic injury, which caused effusion of SF and/or requiring removal of SF/cartilage/synovium during the operation. Participants were excluded if they received treatment elsewhere at the same time or were suffering from an infection or had a non-rheumatic systemic inflammatory disease. The included samples were subjected to different analyses (table 1).

**Table 1:** Patient metadata

Sample type	Disease	Processing	Gender		Average age at surgery (years±SD)	Average BMI±SD
			M	F		
OA	End-stage OA	**All**	**20**	**36**	**66.2±8.3**	**29.2±4.1**
		SF LC-MS	7	13	67.4±5.8	28.1±4.3
		SF LAL	20	36	66.2±8.3	29.2±4.1
		SF LPS bioactivity	10	18	64.8±7.7	28.8±3.8
		SF cell treatment	8	11	67.2±5.6	27.2±2.6
		SF spike	3	2	67.2±8	25.7±1.1
		SF HI pooled	9	10	64.6±8	30.2±3.5
		Synovial membrane model	1	2	66±13.9	27.7±1.8
RA	RA	**All, SF LAL**	**3**	**4**	**61.1±9.32**	**33.56±8.89**
Trauma	ACL tear, meniscal injury	**All**	**31**	**9**	**37.0±12.8**	**25.2±3.5**
		SF LC-MS	15	5	34.6±13.3	24.9±3.8
		SF LAL	31	8	37.0±12.8	25.2±3.5
		SF LPS bioactivity	17	1	36.2±9.9	26.5±3.4
		SF cell treatment	14	5	33.8±12.6	24.9±4.1
		SF spike	5	0	38.6±12.9	26.3±5.2
		SF HI pooled	9	3	37±16.4	25.4±4.8
		Primary chondrocytes	0	1	30	–

ACL, Anterior Cruciate Ligament; BMI, Body Mass Index; LAL, limulus amoebocyte lysate; LC-MS, liquid chromatography–mass spectrometry; LPS, lipopolysaccharides; OA, osteoarthritis; RA, rheumatoid arthritis; SF, synovial fluid.

### Cell treatments

Two cell types were treated with SF; human monocytic leukaemia reporter cell line THP1-Dual-MD2-CD14-TLR4 Cells (Invivogen, USA) and primary human chondrocytes isolated from cartilage collected from a trauma patient. Cells were treated with 1000 ng/mL LPS from *Escherichia coli* B8 (Sigma-Aldrich, USA) and 10% SF with or without 100 µM polymyxin B (Sigma-Aldrich, USA) THP1 cells for 24 hours and chondrocytes for 48 hours. Media was collected and used for QuantiBlue (Invivogen, USA) and QuantiLuc (Invivogen, USA) assays for THP1 cells. The nitric oxide (NO) production by chondrocytes was measured in chondrocyte media using the Griess Reagent kit (Thermo-Fischer, USA). Chondrocyte viability was quantified using CellTiter-Glo 2.0 Cell Viability Assay (Promega, USA). All assays were performed according to the manufacturers’ protocols.

For assessing Fn’s protective capacity, THP1 cells were treated with 1 µg/mL human plasma Fn (Sigma-Aldrich, USA) and Recombinant Human Fn Fragment 3 Protein (R&D Systems, USA) native or previously heat inactivated for 1 hour at 72°C. To understand its interaction with LPS, Fn was either added to the cells at the same time as LPS or premixed and preincubated with LPS for 1 hour at 37°C before treating the cells with the mixture.

### Animal experimentation

The animal study was approved by the Veterinary Office of the Canton Zürich (License No. ZH158/2021) and conducted in accordance with ARRIVE guidelines. Six female Wistar rats (252–324 g, 6 months old, Janvier Labs France) were used in this study. The animals were randomised into the LPS group (three rats) and control group (three rats). Continuous infusion of 300 µg/kg/day *E. coli* LPS B6 (Sigma-Aldrich, USA) for 32 days was conducted to induce endotoxaemia in the LPS group. The total amount of LPS delivered to each animal was 9.6 mg/kg of body weight, which was higher than the 5 mg/kg used in rats as reported by Uchiumi *et al*,^34^ and the 1 mg/kg in mice as reported by Mendez *et al*.^35^ In brief, the animals were anaesthetised with 2.5% isoflurane via inhalation. For the LPS group, an osmotic minipump (Alzet 2ML4, DURECT Corporation, USA) filled with 2 mL LPS prepared in 0.9% NaCl (B. Braun, Germany) was implanted subcutaneously. In the control group, one rat received 0.9% NaCl-filled osmotic pump while the other two remained naïve. Animals were housed in groups of two in individually ventilated cages in a standard laboratory animal environment (21±3°C and 12/12 hour light–dark cycle), received acidified tap water (pH 2.5–3.0) and standard laboratory rodent diet (KLIBA NAFAG, CH) at opportunistic pathogen-free hygiene. Each animal represents an experimental unit. At 1, 3, 5 and 6 weeks after surgery, 0.7 mL blood was collected from the sublingual vein in anaesthetised rats as previously described.^36^ LPS animals were prematurely euthanised at 6 weeks after the surgery due to skin ulcers and control animals were euthanised at 10 weeks postsurgery. After euthanasia, knee joints and joint capsules were harvested and immediately fixed in 4% paraformaldehyde for 1 week. SF and blood plasma were collected as previously described^37^ and stored at −80°C.

### LPS measurements

LPS was measured in samples by three different methods. Liquid chromatography–mass spectrometry (LC-MS) was used for 20 SF samples per group using a patented technology for detection of 3-OH fatty acids as previously described.^38^,^40^ These fatty acids (10:0(3OH), 12:0(3OH), 14:0(3OH), 16:0(3OH) and 18:0(3OH)) are bound to lipid A region of LPS and can be used to identify different bacterial sources. Specifically, 10:0(3OH) is commonly observed in *Bordetella* sp or *Pseudomonas* sp, 12:0(3OH) is found in *Pseudomonas* sp and *Neisseria* sp, 14:0(3OH) is characteristic of Enterobacteriaceae LPS, 16:0(3OH) described in *Porphyromonas* sp, *Prevotella* sp and others, and 18:0(3OH) is observed in LPS from *Francisella* sp and *Helicobacter* sp.

LPS was also measured with Pierce Chromogenic Endotoxin Quant Kit (Thermo-Fischer Scientific, USA) according to manufacturer’s instructions.

We quantified TLR4 activation representing LPS bioactivity from serum samples with HEK-BlueTM hTLR4 SEAP reporter cells (InvivoGen, USA) as previously described.^10^

### 3D synovial membrane model

Synovial membranes from 3 OA donors were digested with 500 U/mL Collagenase I (Sigma-Aldrich, USA) and 200 U/mL DNase I (Stemcell, Canada) in DMEM (Gibco, USA) solution overnight at 37°C. The tissue digest was washed with DMEM and passed through a 70 µm filter (Sarstedt, Germany). Cells were stained (online supplemental table S2) and sorted (online supplemental figure S7) to CD90+PDPN+ synovial-like fibroblasts. The cells were expanded and mixed with Matrigel (Corning, USA) and allowed to mature for 20 days in droplet form. Immature constructs after 7 days were treated with 1000 ng/mL LPS for 6 hours and subsequently fixed and embedded in paraffin blocks. Mature constructs after 20 days were either treated with 1000 ng/mL LPS for 6 hours or left untreated and were also fixed and embedded in paraffin blocks.

### Immunofluorescence staining

For immunofluorescence staining, human, rat and 3D model synovium tissue sections were rehydrated and cooked in sodium citrate buffer (pH6) at 100°C for 60 min for antigen retrieval. They were subsequently blocked with 5% BSA for 1 hour and stained with primary antibodies for Fn (NBP1-91258, Novus Biologicals, USA, 1:400, 2.5 µg/mL) and LPS lipid A (NB100-64484, Novus Biologicals, USA, 1:200, 20 µg/mL) diluted in 1% BSA overnight. The sections were then stained with secondary antibodies anti-goat AF488 (A-11055, Invitrogen, USA, 1:500, 4 µg/mL for all tissues) and anti-rabbit AF647 (A-21244, Invitrogen, USA, 1:500, 4 µg/mL for all tissues) and DAPI (1:1000, Thermo-Fisher, USA) also diluted in 1% BSA for 1 hour. Stained sections were coverslipped with Anti-Fade Fluorescence Mounting Medium (Abcam, UK) and scanned on an Olympus SlideView vs200 device (Olympus, Japan) with 20× magnification within 24 hours and then stored at −20°C for future use. Next, they were exported as .tiff files and analysed using a custom script in QuPath V.0.5.1. provided in supplementary methods.

### Statistical analysis

Data are plotted to represent the mean with 95% CIs with significance indicated with asterisks: *p<0.05, **p<0.01, ***p<0.001, ****p<0.0001. Experiments were independently repeated at least three times. The normality of the data was assessed with the Shapiro-Wilk test. Statistical significance between two groups was assessed using the Mann-Whitney test. When there were more than two groups, one-way analysis of variance (ANOVA) with a Kruskal-Wallis multiple comparisons or two-way ANOVA were used with Tukey’s or Šidak’s multiple comparisons test. Statistical analyses were conducted using GraphPad Prism V.10.4.0.

## Results

### High concentration of LPS with low biological activity is found in human arthritic and trauma knee SF

**Figure 1:**
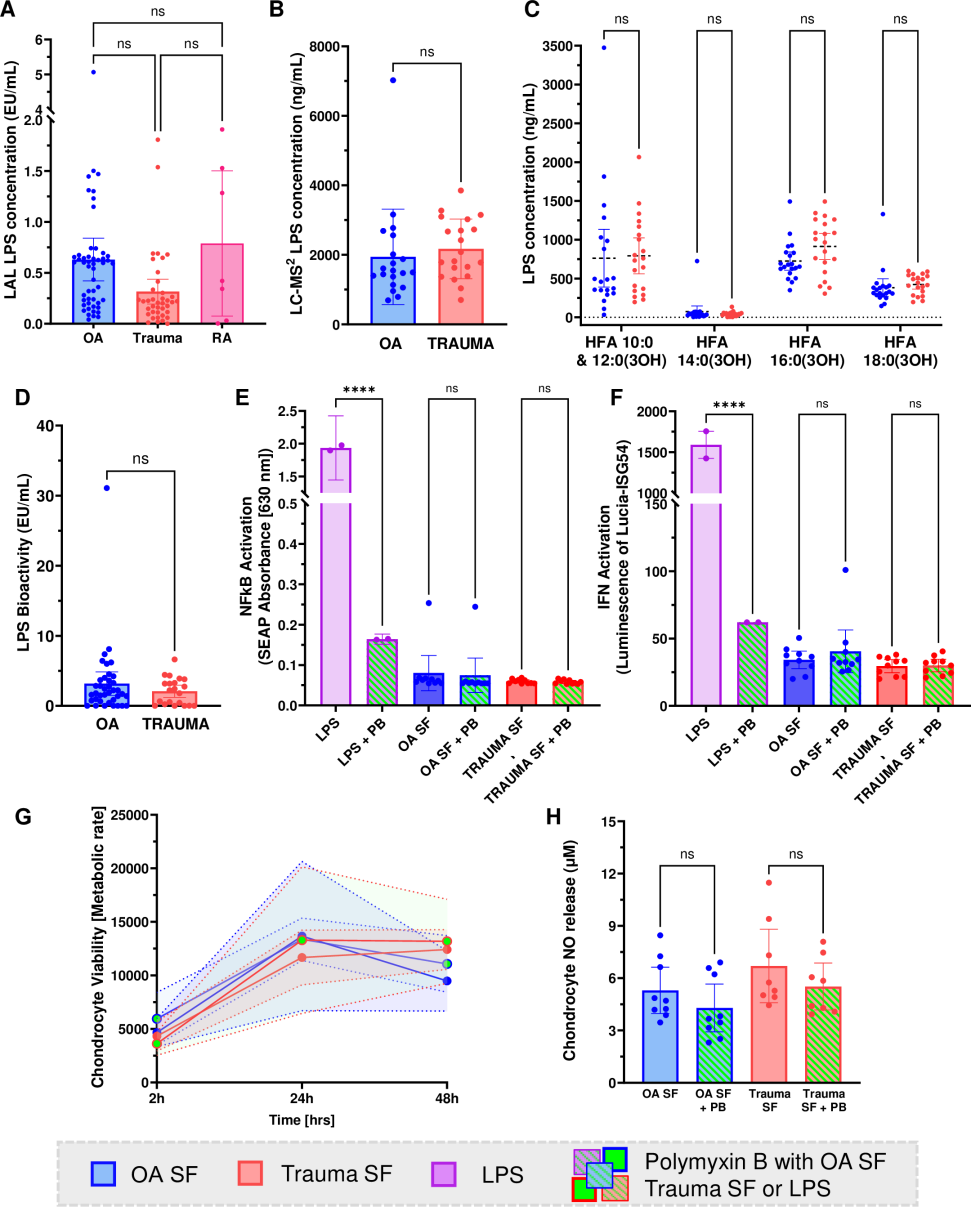
LPS with low inflammatory activity is present in human SF. (A) Pierce Chromogenic Endotoxin Quant Kit analysis of LPS concentration in the human knee SF of trauma (red, n=38), OA (blue, n=49), RA (dark pink, n=7) patients (two-way ANOVA). (B) Total LPS concentration measured with LC-MS in human SF (n=20 per group, Mann-Whitney test) and (C) LPS concentration separated into groups based on Lipid A HFA composition in human SF (n=20 per group, two-way ANOVA). (D) LPS bioactivity in human SF measured with HEK TLR4 reporter cells (OA n=38, trauma n=21, Mann-Whitney test). (E) NFkB activation of LPS in human SF (Kruskal-Wallis test). (F) IFN activation of LPS in human SF (Kruskal-Wallis test). (G) LPS-dependent chondrocyte viability after treatment with human SF. (H) LPS-dependent NO release by chondrocytes after 48 hr treatment with human SF (Kruskal-Wallis test). ANOVA, analysis of variance; LC-MS liquid chromatography–mass spectrometry; LPS, lipopolysaccharides; OA, osteoarthritis; PB, Polymyxin B; RA, rheumatoid arthritis; SF, synovial fluid; HFA, hydroxy fatty acid; NO, nitric oxide; IFN, interferon.

Using LC-MS, we quantified total LPS concentration in knee OA and trauma SF and analysed the types of LPS present based on hydroxy fatty acid chain (HFA) composition of lipid A. LPS concentration was comparable between groups, with OA mean LPS concentration 1942 ng/mL (95% CI 2584.5 to 1299.5) and Trauma mean LPS concentration 2172.3 ng/mL (95% CI 2573.7 to 1770.,9) (figure 1B). None of the included variables (disease, age, sex, body mass index(BMI)) showed a statistically significant association with LPS concentration (p>0.3 for all predictors), suggesting that demographic differences between study groups did not confound the relationship between patient group and SF LPS levels (online supplemental figure S1). The concentration measured using LC-MS was higher than the concentration measured by the standard endotoxin quantification kit (figure 1A,B). No significant difference of LPS concentration was observed between trauma, OA and RA knee SF using standard endotoxin quantification kit (figure 1A). The results of the two techniques did not correlate with each other (online supplemental figures S2 and S3). Lipid A HFA relating to bacterial species of origin was not significantly different between the OA and trauma groups in neither quantity nor composition (figure 1C). Notably, LPS with HFA length of 14:0(3OH), commonly described in *E. coli*, was the least abundant in both groups, whereas HFA 16:0(3OH) and HFA 10:0 and 12:0 (3OH) were the most abundant (figure 1C, online supplemental figure S4). Next, we tested the bioactivity of LPS in SF using HEK TLR4 reporter cells. The cells responded to an amount of LPS that was 1000× lower than LPS quantified with LC-MS (figure 1B,D). There was no difference in LPS bioactivity between OA and trauma SF. To further evaluate the cellular response to SF LPS, we treated THP1 reporter cells with the SF for 24 hours with or without addition of polymyxin B (PB). PB binds and neutralises LPS, to be able to discern the effects of LPS from the effects of other molecules with TLR activation potential in the SF. We did not observe any significant upregulation of NF-κB signalling or interferon activation compared with PB-treated controls, and there was no significant difference between the two patient groups (figure 1E,F). Furthermore, we repeated the same experiment on primary human chondrocytes. SF LPS had no effect on chondrocyte viability and NO release compared with the group treated with SF and PB (figure 1G,H).

### Endotoxemia does not accelerate knee joint degeneration *in vivo*

**Figure 2:**
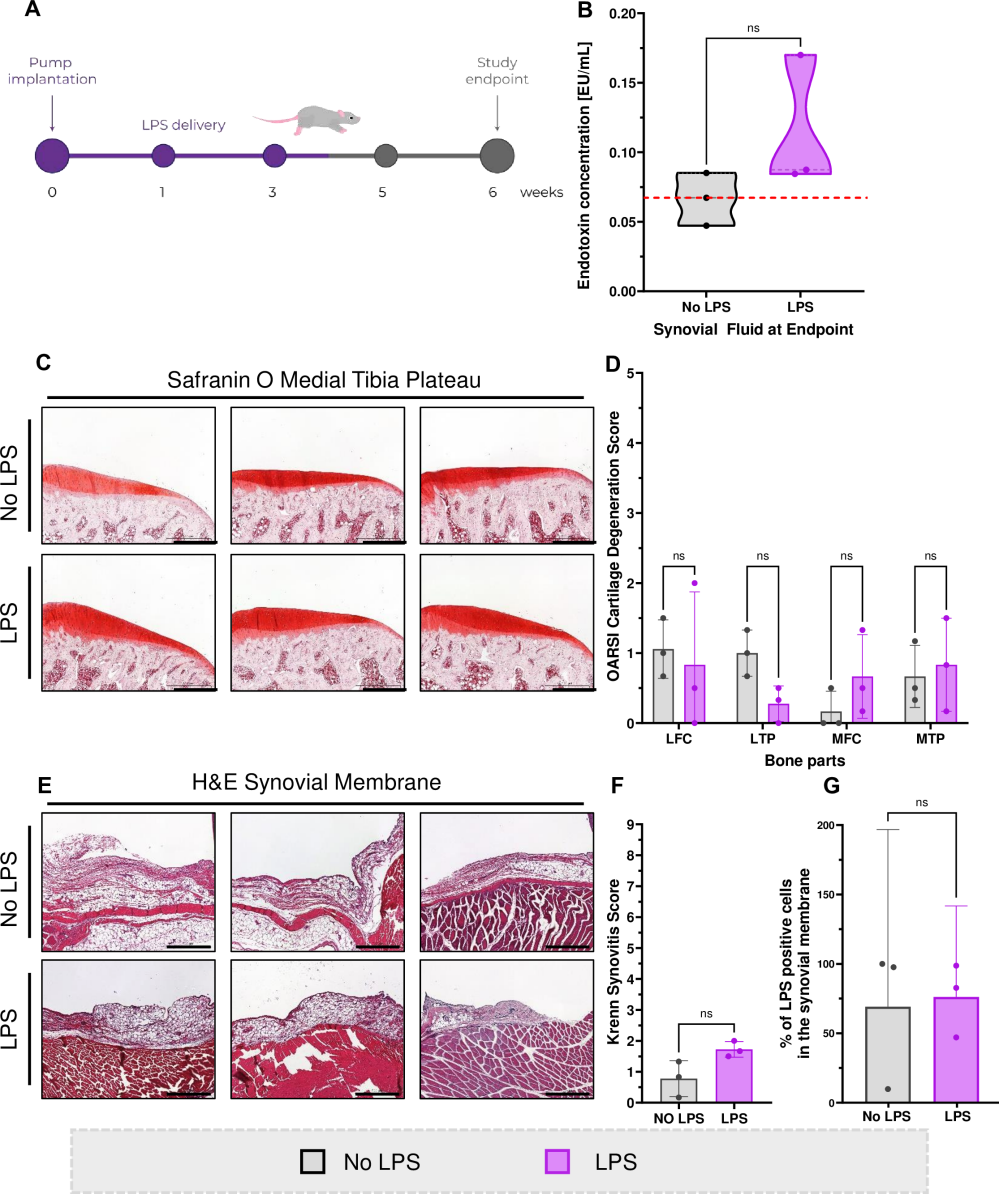
Endotoxemia does not accelerate joint degeneration in vivo: (A) Schematic of the animal experiment. (B) LPS concentration measured in rat SF at endpoint (paired t-test). (C) Safranin O staining of medial tibial plateaus (each image represents one animal) that were graded to allow for (D) the semiquantitative analysis using the OARSI CDS (two-way ANOVA). Scale bar 500 µm. (E) H&E staining of synovial membrane, (each image represents one animal) that were graded to allow for (F) the semi-quantitative analysis using the Krenn Synovitis Score (Mann-Whitney test). Scale bar 500 µm. (G) Semi-quantitative analysis of LPS+ cells in the synovial membrane (paired t-test). Data represent mean + 95%CI. N = 3 per group. ANOVA, analysis of variance; CDS, cartilage degeneration score; LFC, lateral femoral condyle; LPS, lipopolysaccharides; LTP, lateral tibial plateau; MFC, medial femoral condyle; MTP, medial tibia plateau; Ns, not significant.

We subcutaneously implanted pumps to Wistar rats that released LPS for 32 days to induce endotoxemia and investigate its effect on LPS concentration in SF and knee joint degeneration (figure 2A). We collected SF from rat knees and measured LPS concentration. LPS group showed a slight increase in intra-articular LPS concentration (mean: 0.11 EU/mL, 95% CI −0.01 to 0.23) when compared with control group (mean: 0.07, 95% CI 0.02 to 0.11), however, the increase was not statistically significant (p=0.20) (figure 2B), which could indicate that the ability of SF to block LPS is conserved between rats and humans. We embedded and stained the femurs and tibias with Safranin O and histologically graded them using OARSI cartilage degeneration score. There was no observable cartilage degeneration and none of the samples in any of the bone areas exceeded score 2 out of 5 (figure 2C,D). Based on Krenn’s synovitis score, there was no synovitis present in the LPS group (mean: 1.72, 95% CI 1.09 to 2.35) with the score similar to control (mean: 0.78, 95% CI −0.67 to 2.22; p=0.10) (figure 2E,F). We found no difference in the % of LPS+cells in the synovial tissue between the two groups (figure 2G). There were also no osteophytes present in any of the rats. Based on µCT scans, subchondral bone thickness and trabecular bone volume were measured. We found no changes in any of the bone parameters measured in any of the bone areas except for the percentage of bone volume over total volume in the medial tibial plateau (online supplemental figure S7).

### Fn layer in the synovial membrane binds LPS and prevents its migration

**Figure 3:**
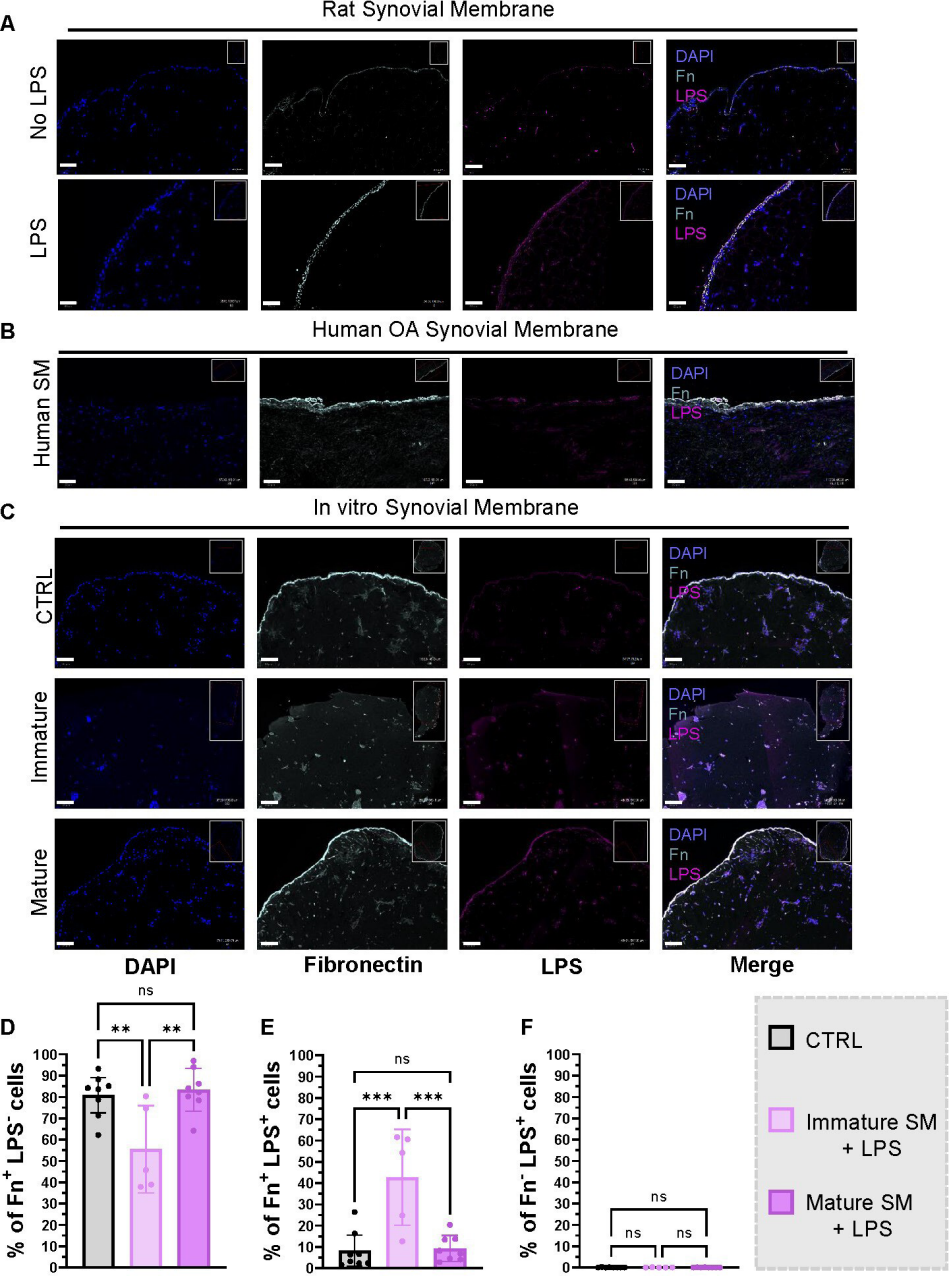
Fibronectin layer in the synovial membrane binds LPS and prevents its migration: Immunofluorescent staining with Fn and LPS of (A) rat SM (B) human OA SM (C) in vitro SM constructs control and immature and mature constructs after treatment with LPS for 6 hours (D–F) semiquantitative analysis of (D) Fn+ LPS− (E) FN+ LPS+ (F) Fn− LPS+ cells in the inner parts of the treated constructs (Ordinary one-way ANOVA). Scale bar 50 µm. ANOVA, analysis of variance; Fn, fibronectin; LPS, lipopolysaccharides; OA, osteoarthritis; SM, synovial membrane.

One of the LPS functions is to provide charges on bacterial outer surface, which influences surface adherence and biofilm formation.^41^ Fn is commonly found in SF and has been implicated in impairing bacterial adhesion to joint prosthetics.^42^ Fn is a ubiquitous molecule that is present in the synovial membrane. We investigated the presence of Fn by performing an immunofluorescence staining of the rat and human OA synovial membrane with LPS and Fn. This revealed the presence of LPS in the synovium and its colocalisation with Fn in the lining layer (figure 3A,B). We did not find any LPS that did not colocalise with Fn (Fn^−^ LPS^+^) in the lining layer, and there was no observed difference between the LPS-treated rats compared with control (online supplemental figure S10). To test the hypothesis that the synovium lining with its Fn layer acts as an LPS barrier, we generated 3D human synovial membrane constructs from synovial-like fibroblast cells isolated from 3 OA patients (online supplemental figure S9 and table S2) and let them mature for 7 or 20 days. We defined day 7 constructs as immature as they did not have an Fn lining layer and day 20 samples as mature as they developed the Fn layer like the one observed in the native synovial membrane (figure 3A,C). We treated the samples with LPS for 6 hours and stained them for LPS and Fn. We found that LPS did not penetrate the constructs with a mature Fn layer, but it could penetrate the immature constructs without Fn (immature vs mature constructs p=0,0005, control vs mature constructs p=0,9821) (figure 3E,F).

### Fn can bind LPS and block its bioactivity

**Figure 4:**
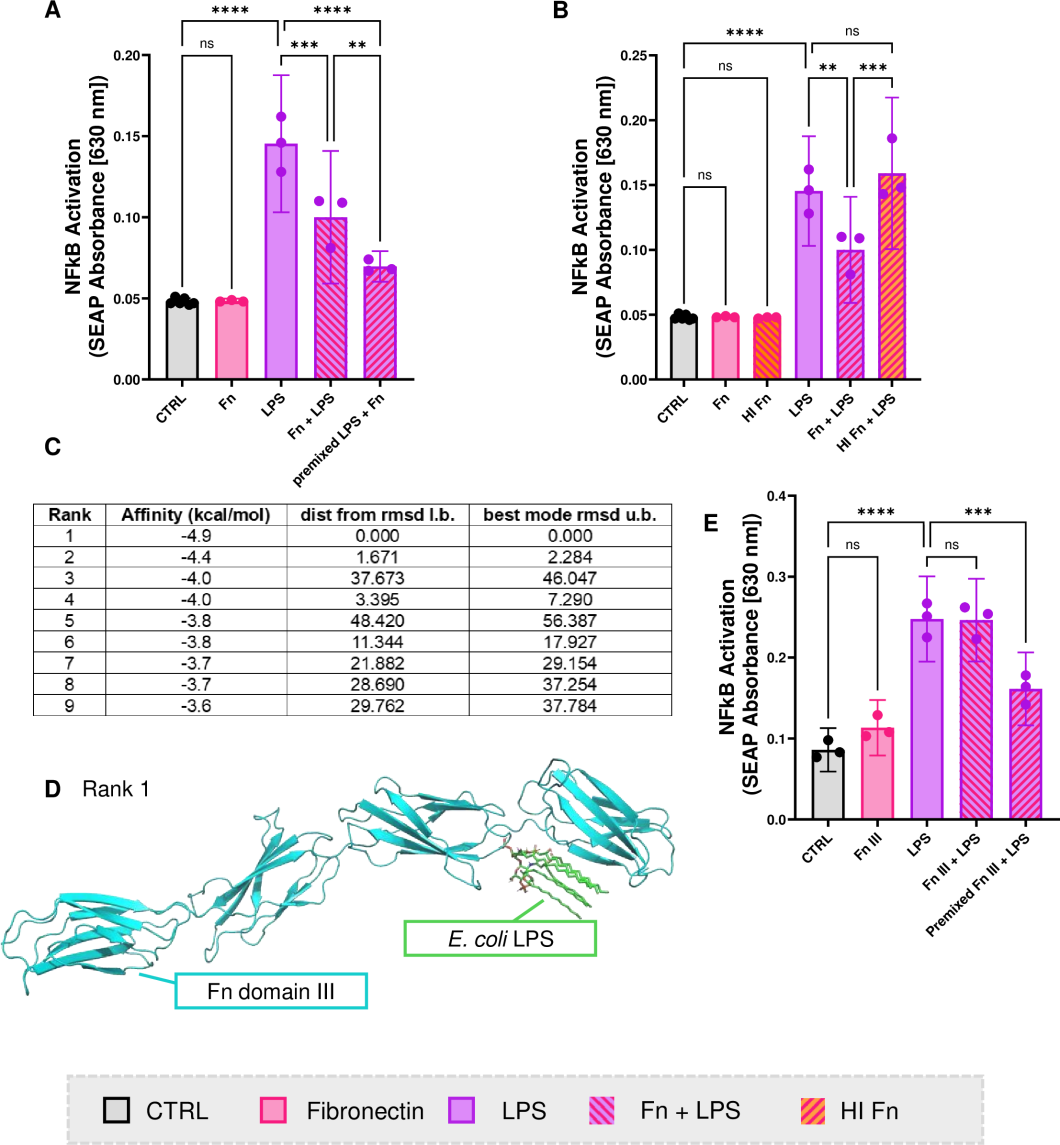
**Fibronectin binds LPS and blocks its bioactivity:** A) THP1 cell NFkB activation of LPS when treated with Fn at once or after premixing LPS and Fn before treatment (1way ANOVA) B) THP1 cell NFkB activation of LPS when treated with native Fn or heat inactivated Fn (1way ANOVA) C) A table summarising the top 9 docking positions between *E. coli* LPS and Fn domain III (Affinity measured by Gibbs free energy, Root Mean Square Deviation (RMSD) of the ligand's position compared to a reference structure, "Dist from rmsd l.b." = distance from RMSD lower bound, "Dist from rmsd u.b." = distance from RMSD upper bound). D) Image of Rank 1 docking position E) NFkB activation of LPS when treated with Fn domain III at once or after premixing LPS and Fn domain III before treatment (1way ANOVA). Fn, fibronectin; LPS, lipopolysaccharides.

To test whether Fn can directly bind LPS, we performed a binding affinity assay by microscale thermophoresis showing that the two molecules can chemically bind (online supplemental figure S6 and table S1). We tested how this binding affects LPS bioactivity in vitro by treating THP1 cells with LPS and Fn. We treated them with both molecules at the same time or by premixing them before adding them to the cells, as some Fn domains are known TLR4 agonists^40^ and we wanted to differentiate between its binding to TLR4 and LPS, respectively. Despite the premixing, Fn could still act as a TLR4 partial agonist. Fn reduced LPS-dependent NF-κB activation in both conditions (LPS 0.14 A, 95% CI 0.1 to 0.18; LPS+Fn 0.1 A 95% CI 0.06 to 0.14; p=0.01) most notably in the premixed condition (0.07 A, 95% CI 0.06 to 0.08, p=0.0004), suggesting that it has the capacity to bind LPS directly (figure 4A). We further showed that heat inactivation of Fn reduced the ability of Fn to reduce LPS-dependent NF-κB activation in THP1 cells, suggesting the binding is sterical (figure 4B). To investigate the interaction between LPS and Fn we performed an in silico analysis to predict the location of binding. The analysis showed that *E.coli* B6 LPS binds Fn domain III between the subunits, notably, the Gibbs free energy is −4.9 kcal/mol for the most favourably ranked position (figure 4C,D, online supplemental figure S11). We further confirmed that Fn domain III can functionally bind LPS by showing that Fn domain III alone reduces LPS-dependent NF-κB activation (p=0,0002) of THP1 cells when premixed with LPS (figure 4E).

## Discussion

In this study, we found that LPS concentration in SF is comparable between arthritis disorders (OA, RA) and trauma (figure 1A,B), suggesting that LPS is naturally present in SF. Analysis of Lipid A HFA composition with the help of LC-MS demonstrated that the composition is the same between trauma and OA (figure 1C), indicating that bacterial phyla of LPS origin as well as LPS modification processes are similar between the groups. Highly immunogenic lipid A attributed to *E. coli* (HFA 14:0(3OH)) was present at the lowest concentration in both groups, which supports our in vitro data showing that LPS in SF has low biological activity (figure 1D). Interestingly, LPS levels measured with LC-MS were substantially higher than previously reported levels measured by the endotoxin quantification assay^12^ as well as measured in the current study with the same assay (figure 1B). Suggesting that the total amount of LPS present in the human SF is very high, however, it is modified and blocked from being sensed at the same concentration neither by the cells nor by the enzymes in the endotoxin quantification assay. The two assays are reported in different units. To facilitate comparison, it is important to note that 1 EU/mL is approximately equivalent to 0.1–0.2 ng/mL, according to FDA recommendations.^43^ It is important to note that standard endotoxin quantification kits are based on the limulus amoebocyte lysate (LAL) assay, and it is currently unknown how LPS sensing by the coagulation enzymes translates to physiological relevance. These enzymes cannot measure all LPS types and react with varying intensity to different LPS, therefore, LAL is not a precise method for LPS measurement^44^ (online supplemental figures S1 and S3). Furthermore, LAL failed in the diagnosis of Gram-negative sepsis, because it reacted to B-glucan instead of LPS.^45^ This contradictory data lead to the fact that to this day, FDA has not yet accepted the LAL test for blood endotoxin detection.

Interestingly, LPS bioactivity assay measured 1000 x lower concentrations of LPS compared with LC-MS (figure 1A,D). Low bioactivity is caused by known processes such as liver enzymatic LPS digestion^21^ and blocking by the plasma factors.^46^ Our data show that SF also retains the capacity to block LPS (figure 1A–F). We measured bioactivity with the help of HEK TLR4 reporter cells and studied the cellular response with wildtype THP1 cells that give us a broader image of the cellular response. There was a lack of NF-κB and interferon response to SF LPS by THP1 cells (figure 1E,F). Furthermore, SF capacity to block LPS detection and inflammatory capacity were impaired after heat inactivation of SF (online supplemental figure S5). This suggests that a heat-sensitive protein is masking or blocking LPS from detection. Combined with the lack of strong immune response to native SF samples, this indicates a protein-mediated LPS masking effect of synovial protein(s) present in the SF and shows the capacity of human SF to block the inflammatory effects of LPS.

In line with these results, our in vivo study demonstrated that induced endotoxaemia does not accelerate OA-like knee joint destruction of healthy rats. The data suggest that LPS was neutralised, and its bioactivity impaired, as only a small increase in LPS concentration was observed in the plasma and the SF of the treated group compared with control (figure 2B, online supplemental figure S6). Furthermore, the LPS-treated group did not develop OA-like symptoms, as neither cartilage nor the synovium were significantly affected (figure 2C–G). The clinical scores of both groups represent non-degenerated joint tissue; however, it should be noted that the power of the statistical analysis is low due to the small number of animals. This study also does not exclude the possibility that there might be exacerbation of underlying inflammation by LPS in a damaged joint.

By further investigating LPS blocking factors, we discovered that LPS colocalised with Fn in the rat synovial membrane. This was confirmed in the human OA synovial membrane (figure 3B). In a 3D synovial membrane model, we found that the Fn-rich layer of the synovial lining may act as a barrier to LPS migration (figure 3C–F) and may reduce its proinflammatory potential, possibly through sequestration or indirect interaction with LPS (figure 4A–D, online supplemental figures S10 and S11). LPS Fn colocalisation likely does not strongly affect synovial cell function, as the proinflammatory stimulation of monocytes treated with premixed LPS Fn complexes was substantially lower compared with stimulation with LPS alone (figure 4A). The novel protective function of Fn reported here is a surprising finding. As Fn has been associated with pathogenesis in OA in its fragmented form.^47^ Fn in its fragmented form might have a lowered capacity to reduce LPS-mediated NFkB activation as is suggested by figure 4E where Fn domain III alone does not reduce NFkB activation without preincubation with LPS. However, it has also been shown that Fn plays a role in bacterial infection,^27^ suggesting its connection with LPS. We postulate that Fn in SF^26^ may act as one of the factors that contribute to LPS sequestration and reduce its proinflammatory potential. We hypothesise that LPS binds the available Fn in the synovial membrane until it reaches a plateau, therefore there might be a dose-dependent relationship on the protective effect of Fn in the tissue. One cannot disregard the possibility that LPS binding to Fn EDA, a known TLR4 agonist, could facilitate or exacerbate its proinflammatory activity. It is also possible that LPS could induce transcription of Fn itself; however, in our rat model, we did not observe a higher expression of Fn in the endotoxemia group. It has previously been shown that LPS can stimulate the production of an Fn receptor, which can facilitate monocyte migration into the tissue and binding to matrix Fn.^48 49^ LPS translocates to the joint from the gut mediated by intestinal permeability.^50^ This mechanism has been reported to play a role in OA, especially metabolic OA.^9 51^ Therefore, the connection between Fn and LPS could influence the gut-joint axis, as Fn is abundant in gut epithelium and reportedly influences intestinal permeability through its connection with CD137 and CD36.^52 53^ Therefore, a disruption in Fn expression could influence its protective role in the gut as well as in the joint. We hope this work inspires more studies into the complex biological relationship between LPS and Fn.

The limitations of this study include the lack of a healthy human SF. This would help us understand whether LPS influx is a process facilitated by injury and degeneration or is a common process present in healthy joints. Using trauma samples as a control is suboptimal due to demographical differences, however, they are not chronically inflamed and the confounding factors had no statistically significant effect on LPS concentration in SF (online supplemental figure S1). Another limitation is the method of LPS quantification. We have compared two published methods of LPS quantification and observed significantly different results between them. This opens the question of an appropriate method and hopefully inspires more studies to carefully select it.

Overall, our findings suggest that high-molecular weight Fn can bind LPS and may contribute to limiting its inflammatory effects, which we propose as a potential novel protective mechanism in synovial joints. We also hope to inspire further research into this connection, as it is possible that there is an interplay between LPS and Fn production, which could further elucidate the role of these factors in the exacerbation of rheumatic diseases associated with altered gut permeability and endotoxaemia.

## Supplementary material

10.1136/rmdopen-2025-005622 online supplemental file 1

## Data Availability

Data are available in a public, open access repository.
