# Supplementary material for: Evaluating the role of lipopolysaccharides in the joint: fibronectin as a novel protective mechanism
Source: RMD Open. 2025 Jul 10;11(3):e005622. doi: 10.1136/rmdopen-2025-005622 (PMC12248214; doi:10.1136/rmdopen-2025-005622)
Supplement: online supplemental file 1 [file rmdopen-11-3-s001.pdf]

1. Supplementary Information

Evaluating the role of lipopolysaccharides in the joint: Fibronectin as a novel protective mechanism

Kajetana Bevc[1], Shipin Zhang[1], Andres Pazos-Perez[2], Ana Alonso-Perez[2], David Fercher[1], Sami Kauppinen[3], Tuomas Frondelius[3], Valentino Bruhin[4], Gian Salzmann[4], Thomas Rauer[5], Christopher Pape[5], Mikko Finnilä[3], Caroline Ospelt[6], Rodolfo Gomez[2], Kari K Eklund[7-8] , Marcy Zenobi Wong[1], Goncalo Barreto[7]

[1]Tissue Engineering + Biofabrication Laboratory, Department of Health Sciences and Technology, ETH Zürich, Otto-Stern-Weg 7, 8093 Zürich, Switzerland

[2]Health Research Institute of Santiago de Compostela, Santiago Clinic Hospital, s/n A, Tr.ª da Choupana, 15706 Santiago de Compostela, Spain

[3]Research Unit of Health Sciences and Technology, University of Oulu, Aapistie 5A, 90220, Oulu, Finland

[4]Schulthess Klinik, Department for Knee Surgery, Lengghalde 2, 8008 Zürich, CH

[5]Department of Traumatology, University Hospital Zurich, Rämistrasse 100, 8091 Zürich, CH

[6]Center of Experimental Rheumatology, Department of Rheumatology, University Hospital Zurich, University of Zurich, Rämistrasse 100, 8091 Zürich, CH

[7]Clinicum, Faculty of Medicine, University of Helsinki and Helsinki University Hospital, Haartmaninkatu 8, 00290, Helsinki, Finland

[8]Department of Rheumatology, Helsinki University Hospital and Helsinki University, Haartmaninkatu 4 00029 HUS, Helsinki, Finland

E-mail: goncalo.barreto@helsinki.fi

Supplementary Material

Supplementary Methods

1. Blinding
2. Sample collection & Isolation of Primary Cells
3. LPS Spike and Heat Inactivation of SF
4. Micro-Computed Tomography (μCT) Evaluation
5. Histology Staining Protocols

6. Binding Affinity

Supplementary Figures & tables

Figure S1 A) Forest plot of regression coefficients with 95% confidence intervals for group, age, sex, and BMI from a linear model predicting synovial fluid LPS levels. All predictors were non-significant, as confidence intervals included zero. Ns= non-significant B-E) Diagnostic plots for multiple linear regression assessing confounding effects of age, sex, and BMI on LPS concentration. To evaluate potential confounding by demographic variables, a multiple linear regression was performed with total LPS concentration as the dependent variable and group (OA vs. trauma), age, sex, and BMI as independent variables in R studio v 1.4.1564. Regression coefficients and 95% confidence intervals were used to assess statistical significance. A forest plot was generated to visually summarize the effect sizes and uncertainty for each covariate.

Figure S2 Pierce™ Chromogenic Endotoxin Quant Kit analysis of LPS concentration and LC-MS LPS concentration linear fit

Figure S3 Pierce™ Chromogenic Endotoxin Quant Kit analysis of LPS concentration and LC-MS LPS concentration correlation plot

Figure S4 Pooled comparison of abundance of LPS in OA and Trauma patient SF with different HFA chain lengths. 1way ANOVA **** <0,0001 *** 0,0001 ns = not significant, n=40

Figure S5 The effect of heat inactivation on LPS concentration in the SF. A,B,E,F) LPS concentration in SF measured with Pierce Chromogenic Endotoxin Quantification kit in human trauma and OA SF A,B,F) before and after LPS spike of 1000 ng/mL and E,F) in pooled samples before and after heat inactivation. C) THP1 cell NFkB response after treatment with native and 1000 ng/mL spiked SF D) Primary chondrocyte NO release after treatment with native and 1000 ng/mL spiked SF. 2way ANOVA.

Figure S6 LPS concentration in the rat plasma

Figure S7 Rat bone analysis

Figure S8 Microscale Thermophoresis analysis of Fibronectin and LPS binding affinity

Table S1 Kd of binding affinity between Fibronectin and LPS measured with Microscale Thermophoresis

Table S2 Synovial like fibroblast Sorting panel

Figure S9 Synovial like Fibroblasts Sorting strategy

Figure S10 Quantification of Fibronectin and LPS single and double positive cells in the rat synovium

Figure S11 *In silico* prediction of binding of LPS and Fn domain III ranks 2-9

Supplementary methods

1. Blinding

Researchers were blinded throughout the study and data analysis with the different groups only being disclosed after the conclusion of the study. Randomly generated numbers were used as unique identifiers for human samples. Animal numbers were used as unique identifiers during the tissue characterization.

2. Sample collection & Isolation of Primary Cells

SF samples were collected during knee surgery and transported to the laboratory where they were centrifuged for 10 minutes at 500 g. The supernatant was collected, aliquoted and transferred to polystyrol endotoxin free tubes (Ficher Scientific, USA) and stored at –80°C until analysis. In addition, synovial membrane tissues and cartilage samples were collected from some patients during the surgery, stored in DMEM (Gibco, USA) supplemented with 5% FBS (Gibco, USA) and transported to the laboratory. Synovial membranes were fixed in 4% paraformaldehyde for one day, then dehydrated in a Logos J device (Milestone, Italy) for subsequent paraffin embedding and microtomy. The cartilage tissue was used for primary chondrocyte isolation. In brief, the cartilage tissue was cut into small pieces using scissors, followed by digestion in 2mg/ml collagenase I (Sigma-Aldrich, USA) dissolved in growth media (DMEM-F12 (Gibco, USA) supplemented with 10% FBS, 1% Pen-strep (Thermo-Fischer, USA) and 50µg/ml ascorbic acid (TCI, Japan)) overnight in at 37°C in a 5% CO_2 incubator. The dissociated cells were passed through a 70µm cell strainer and subsequently cultured in growth media at 37°C in a 5% CO_2 incubator until further use.

3. LPS Spike and Heat Inactivation of SF

SF was spiked with 1000 ng/mL LPS and incubated for 24 hours at 37°C. After incubation LPS concentration was measured with Pierce™ Chromogenic Endotoxin Quant Kit (Thermo-Fischer, USA) and THP1 cells and human primary chondrocytes were treated with 10% non-spiked SF or 10% spiked SF. Cell culture media were collected, and QuantiBlue and the NO assay were performed to assess the responses of THP1 cells and chondrocytes respectively.

SF from several patients was also pooled and heat inactivated (HI) for 1 hour at 70°C, after it cooled to room temperature LPS concentration was measured with Pierce™ Chromogenic Endotoxin Quant Kit.

4. Micro-Computed Tomography (µCT) Evaluation

The rat knee joints were fixed for 7 days in 4% formaldehyde, dehydrated and stained with 1% phosphotungstic acid (in 70% EtOH, Sigma-Aldrich, USA) for 24 hours. They were scanned with a µCT 45 device (Scanco Medical, CH) at 55 kV, 72 µA and 4W using an 0.5 mm aluminium filter (4.5 µm voxel size). µCT evaluation was performed by a blinded experimenter. From the CT dataset a bone tissue binary mask was extracted, and the local subchondral bone plate thickness measured and averaged through the entire condyle with BoneJ in Fiji ImageJ v1.51n. To analyse the trabecular bone, the medullary volume was isolated by identifying the growth plate and the subchondral bone. Next, an ROI was created for the entire volume eroding it with a 315 µm circular kernel. Trabecular bone parameters were calculated with CTAn 1.20.3.0 (Bruker, USA).

5. Histology Staining Protocols

After microCT analysis, rat knee joints and joint capsules were decalcified in aqueous 10% NH4-EDTA solution for 2 weeks. Following decalcification, the samples were dehydrated on a Logos J device and embedded in paraffin. Serial sections were cut (joint capsule: 5 µm transverse, femur/tibia: 5 µm coronal, Microm, GER) and rehydrated for histological staining. Joint capsules were stained with haematoxylin and eosin (HE) for general morphology and scored with Krenn's synovitis score[60] to assess the inflammation status. Distal femur and tibia were stained with safranin O staining (Saf-O) as described in supplementary methods to access the general morphology and sulphated glycosaminoglycan (s-GAG) deposition. The cartilage degeneration score and osteophyte score[61] were performed on Saf-O stained distal femur and tibia sections to semi-quantify the cartilage damage and osteophyte formation. All histological scoring was performed by three blinded independent observers.

5.1 Haematoxylin & Eosin Staining

Rehydrated tissue sections were stained with Gill No. 3 haematoxylin (Merck) Mayer's haematoxylin for 85 min, washed with water, followed by blueing in 0.3% 0.1% Na_2CO_3 for 40 seconds 95% ethanol, and counterstained in 0.25% Eosin Y solution (in acidified ethanol) for 1 minutes 45 seconds. After a brief rinsing in 100% EtOH, the sections were dehydrated in Xylene (2x 1 min each), and cover slipped using Eukitt mounting medium.

5.2 Safranin O

After µCT analysis, rat femur and tibia were decalcified in aqueous 10% NH4-EDTA solution and prepared for histology analogously as above. 5 µm anterior and posterior coronal sections were prepared from the weight bearing area with a spacing of 200 µm. The rehydrated sections were

stained with 0.5% Safranin O for 16 minutes, washed with water for 10 minutes, dehydrated to xylene, cover slipped and scanned on an Aperio AT2 (Leica Biosystems, GER).

5.3 Histology Quantification Macros

All automated histological quantification on the immunofluorescence-stained synovia was run on QuPath v0.5.1 using the macros below. Prior to the analysis, the ROI was defined.

```
setChannelNames("DAPI", "Fn", "LPS");

selectAnnotations();

runPlugin('qupath.imagej.detect.cells.WatershedCellDetection',
'{"detectionImage":"DAPI","requestedPixelSizeMicrons":0.5,"backgroundRadiusMicrons":8.0,"
backgroundByReconstruction":true,"medianRadiusMicrons":0.0,"sigmaMicrons":1.5,"minArea
Microns":10.0,"maxAreaMicrons":400.0,"threshold":100.0,"watershedPostProcess":true,"cellEx
pansionMicrons":5.0,"includeNuclei":true,"smoothBoundaries":true,"makeMeasurements":true}'
);

runObjectClassifier("Construct Fn v1","Construct LPS v1");

def name = getProjectEntry().getImageName() + '.txt'

def path = buildFilePath(PROJECT_BASE_DIR, 'annotation results')

mkdirs(path)

path = buildFilePath(path, name)

saveAnnotationMeasurements(path)

print 'Results exported to ' + path
```

6. Binding Affinity

Binding was confirmed and measured by MicroScale Thermophoresis (MST) with a Monolith device (Nanotemper, Germany). For this Fn was first labelled with Atto 647 NHS ester kit (Sigma-Aldrich, USA) and it’s degree of labelling measured at 1 as required. Labelled Fn was mixed in MST buffer (150 mM NaCl, 10 mM MgCl2, 50 mM TrisHCl, 0.15% Tween20 (Sigma-Aldrich, USA) with LPS B8 at 16 different concentrations starting with 2.5 mg/mL and incubated for 1hr at 37°C . The mixtures were then loaded to Monolith Premium Capillaries (Nanotemper, Germany). Binding affinity was measured at 37°C at a low IR laser setting. The Kd was calculated by the MO.Control V2.6.3 based on the curve fit.

A molecular docking analysis was used to get a computational approximation of the strength of interaction between two proteins or protein molecules. Fibronectin domain III and LPS *E. coli* B6 structures were first optimized using PyMol software 2.5.228. Easy Dock Vina 2.2 was used for the molecular docking analysis[62]. Results are shown in Gibbs free energy units (Kcal/mol) and

ranked from lowest (strongest interaction) to highest (weakest interaction) for all the possible conformations.

Supplementary figures and tables

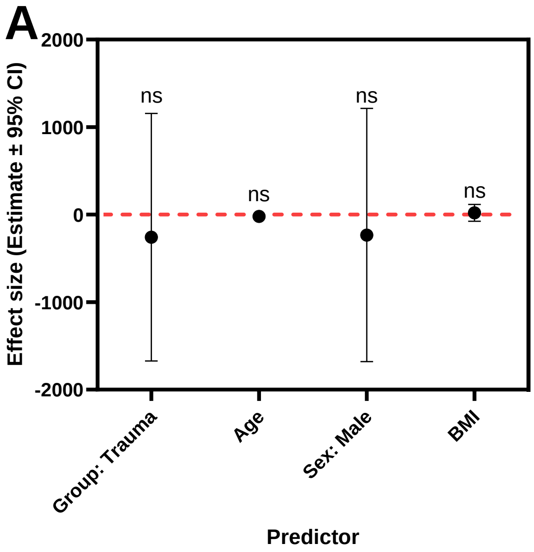

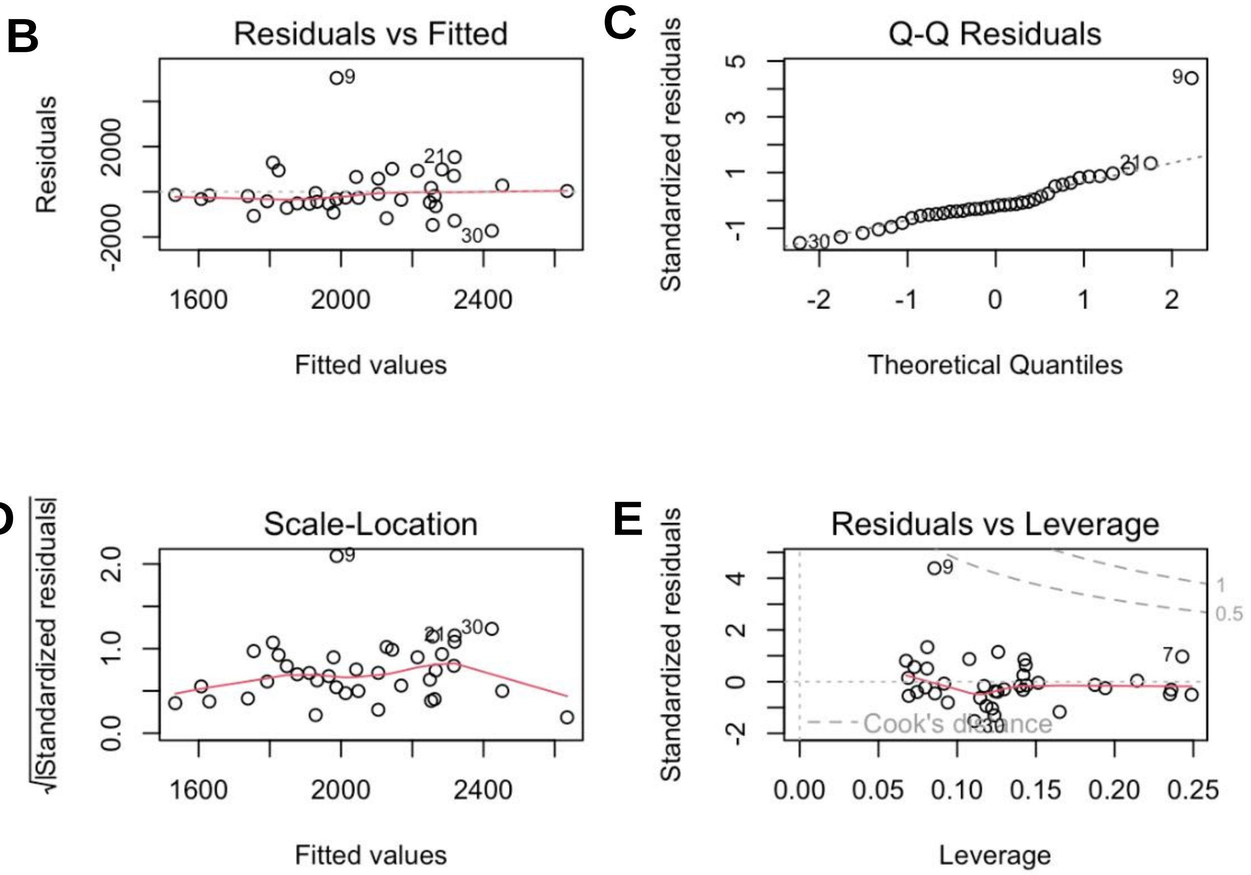

Figure S1 A) Forest plot of regression coefficients with 95% confidence intervals for group, age, sex, and BMI from a linear model predicting synovial fluid LPS levels. All predictors were non-significant, as confidence intervals included zero. Ns= non-significant B-E) Diagnostic plots for multiple linear regression assessing confounding effects of age, sex, and BMI on LPS concentration. To evaluate potential confounding by demographic variables, a multiple linear regression was performed with total LPS concentration as the dependent variable and group (OA vs. trauma), age, sex, and BMI as independent variables in R studio v 1.4.1564. Regression coefficients and 95% confidence intervals were used to assess statistical significance. A forest plot was generated to visually summarize the effect sizes and uncertainty for each covariate.

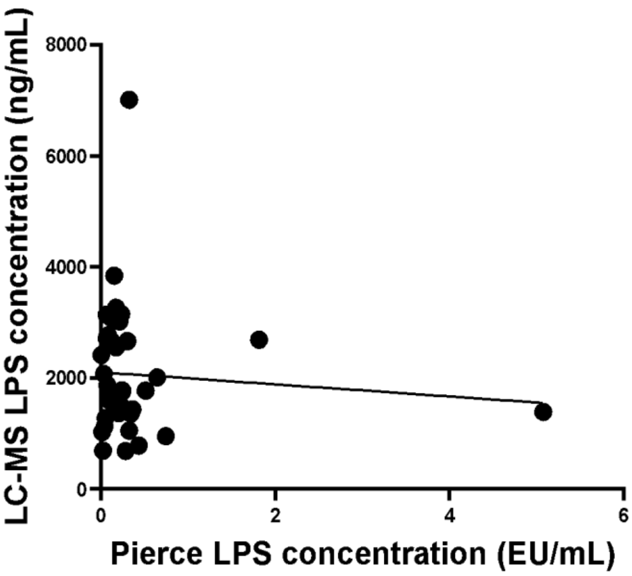

Figure S2 Pierce™ Chromogenic Endotoxin Quant Kit analysis of LPS concentration and LC-MS LPS concentration linear fit

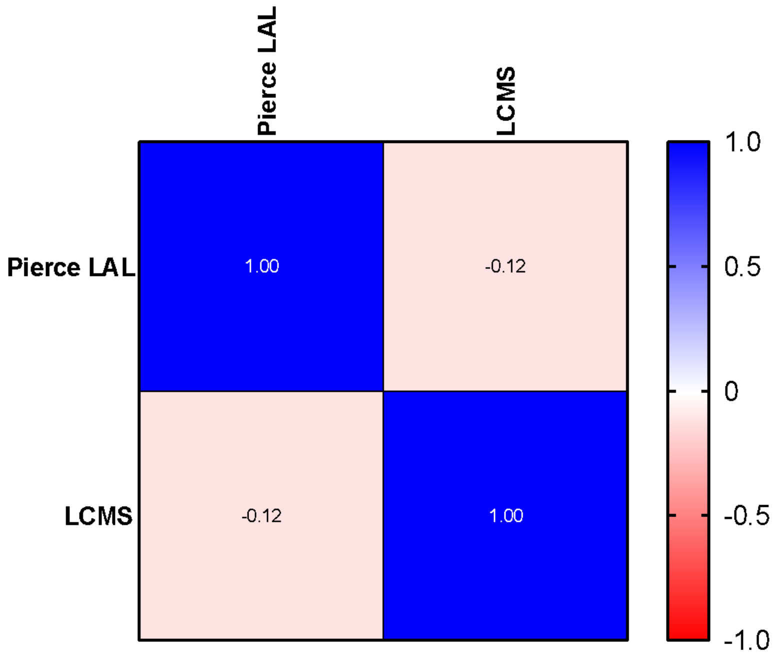

Figure S3 Pierce™ Chromogenic Endotoxin Quant Kit analysis of LPS concentration and LC-MS LPS concentration correlation plot

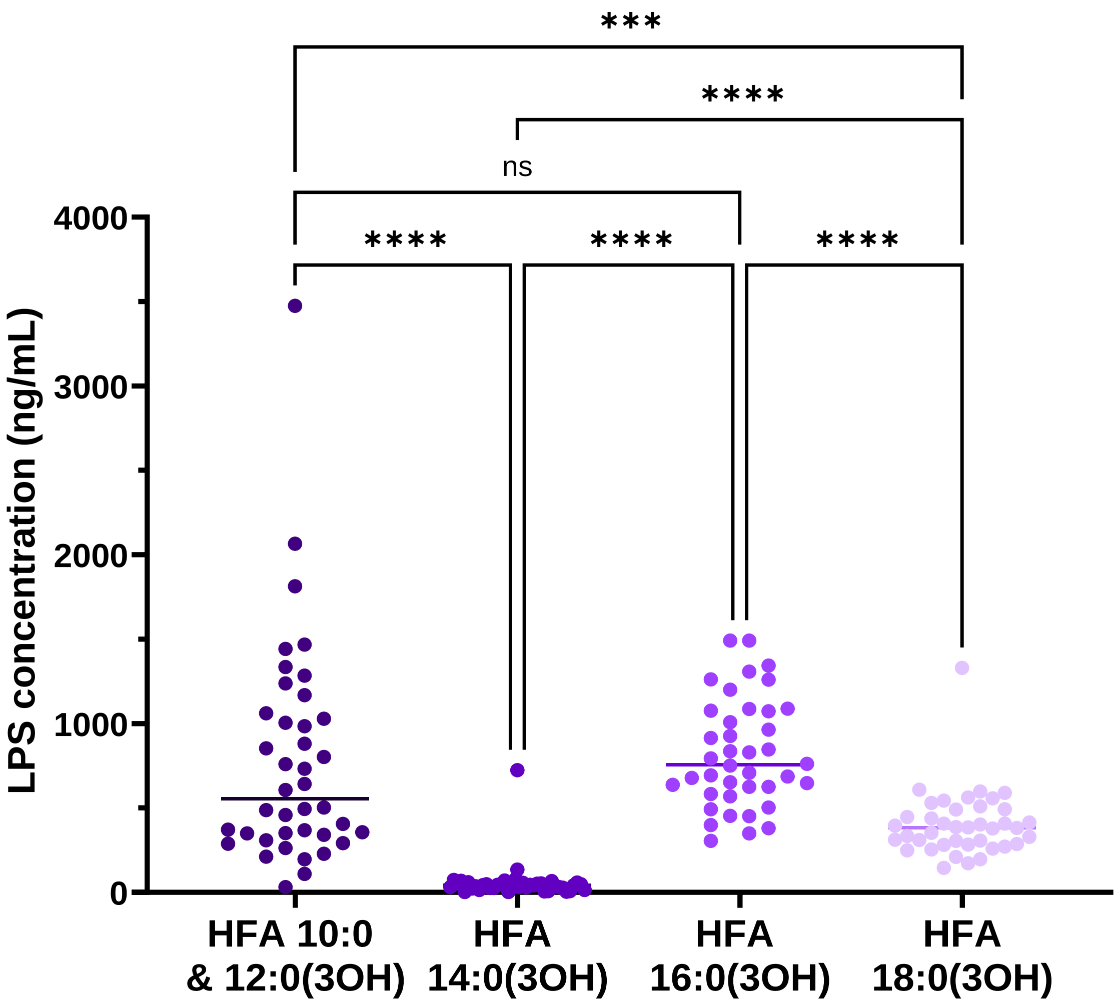

Figure S4 Pooled comparison of abundance of LPS in OA and Trauma patient SF with different HFA chain lengths. 1way ANOVA **** <0,0001 *** 0,0001 ns = not significant, n=40

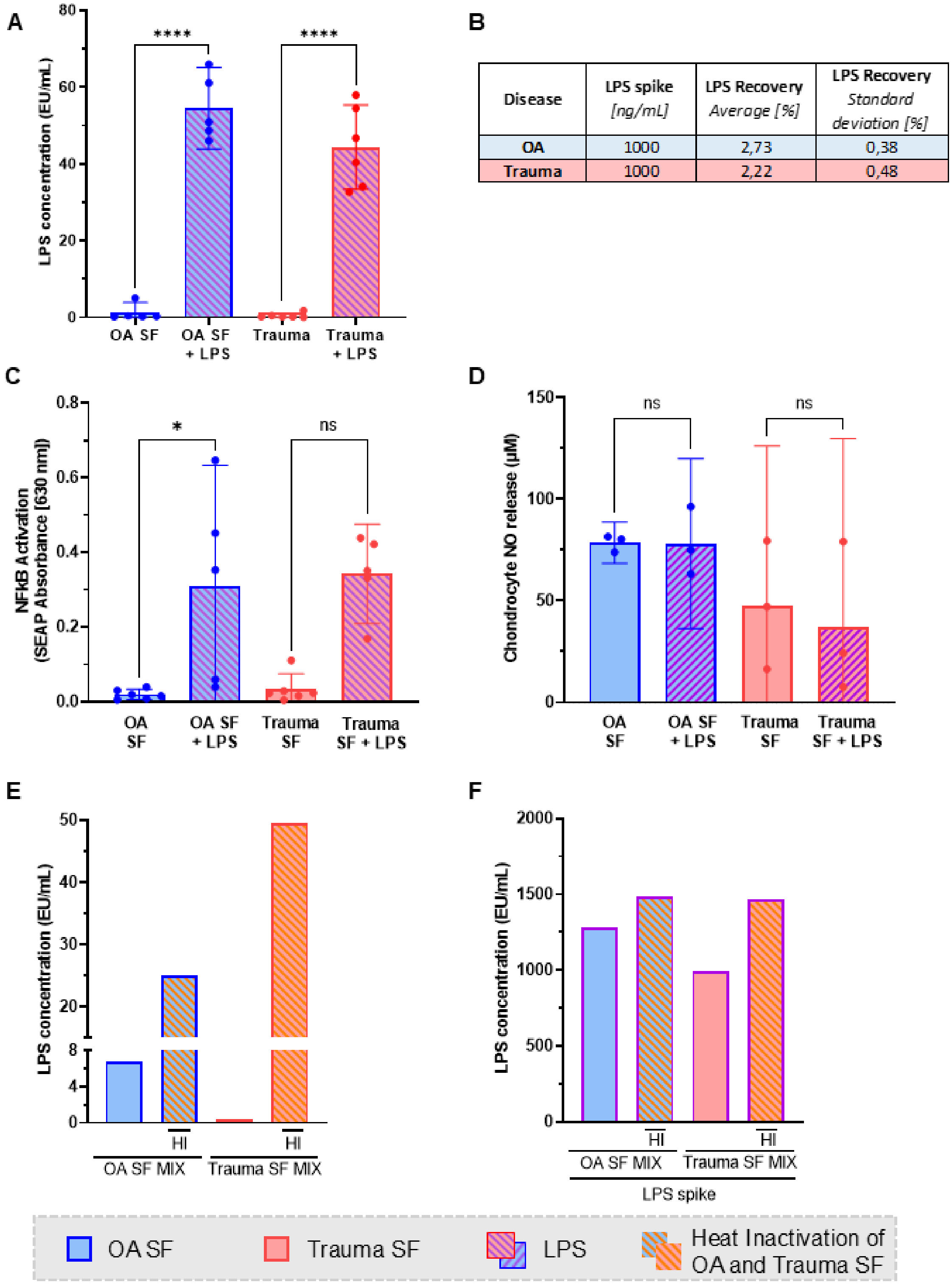

Disease	LPS spike [ng/mL]	LPS Recovery Average [%]	LPS Recovery Standard deviation [%]
OA	1000	2,73	0,38
Trauma	1000	2,22	0,48

Figure S5 The effect of heat inactivation on LPS concentration in the SF. A,B,E,F) LPS concentration in SF measured with Pierce Chromogenic Endotoxin Quantification kit in human trauma and OA SF A,B,F) before and after LPS spike of 1000 ng/mL (equivalent to 2000 EU/mL)

and E,F) in pooled samples before and after heat inactivation. C) THP1 cell NFkB response after treatment with native and 1000 ng/mL spiked SF D) Primary chondrocyte NO release after treatment with native and 1000 ng/mL spiked SF. 2way ANOVA.

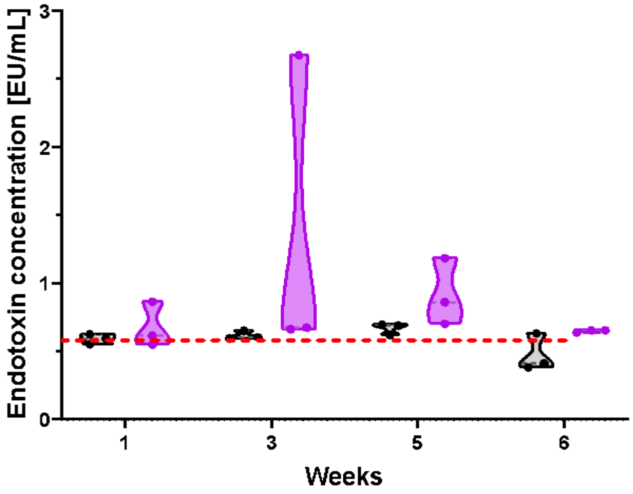

Figure S6 LPS concentration in the rat plasma

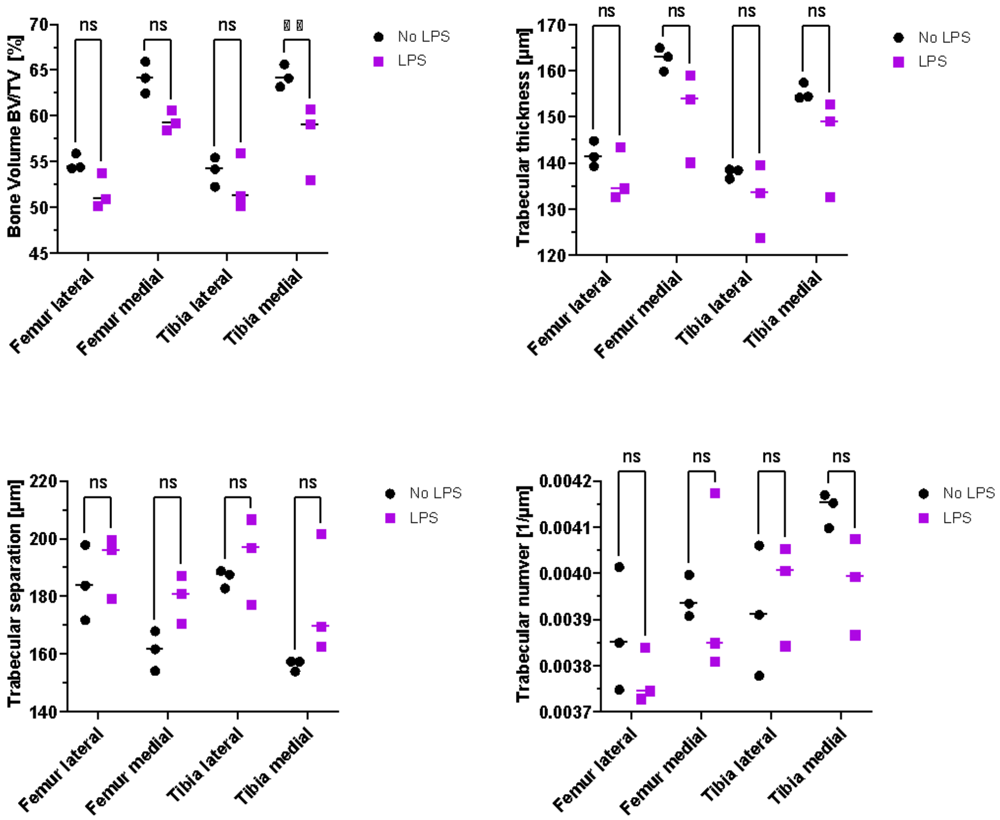

Figure S7 Rat bone analysis

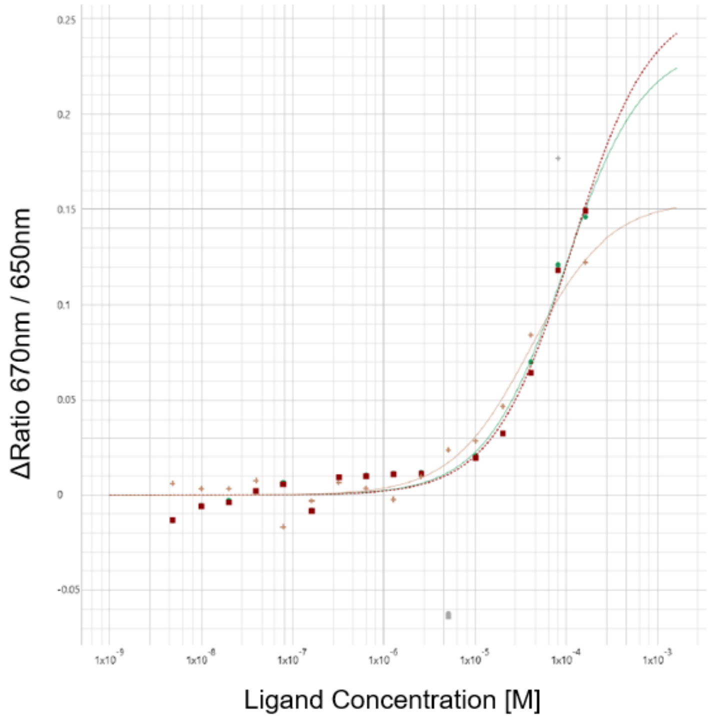

Figure S8 Microscale Thermophoresis analysis of Fibronectin and LPS binding affinity

Table S1 Kd of binding affinity between Fibronectin and LPS measured with Microscale Thermophoresis

Trial	Target (40 nM)	Ligand	Capillary	T [ºC]	Kd [µM]	S/N Ratio
1	Fibronectin	LPS	Premium	37	53.8	31.02
2	Fibronectin	LPS	Premium	37	64.7	35.39
3	Fibronectin	LPS	Premium	37	40.6	23.40

Table S2 Synovial like fibroblast Sorting panel

Marker	Dye	Clone	Brand	Dilution
**CD90**	BV510	5E10	BioLegend #328126	1:80
**CD14**	BV650	M5E2	Biolegend #301836	1:40
**CD206**	BV785	15-2	BioLegend #321142	1:80
**CX3CR1**	FITC	2A9-1	BioLegend #341605	1:80
**CD31**	PE	WM59	BioLegend #303105	1:640
**CD45**	PE-Cy7	HI30	Invitrogen #25045942	1:100
**Podoplanin**	AF647	LpMab-21	BioLegend #395004	1:80

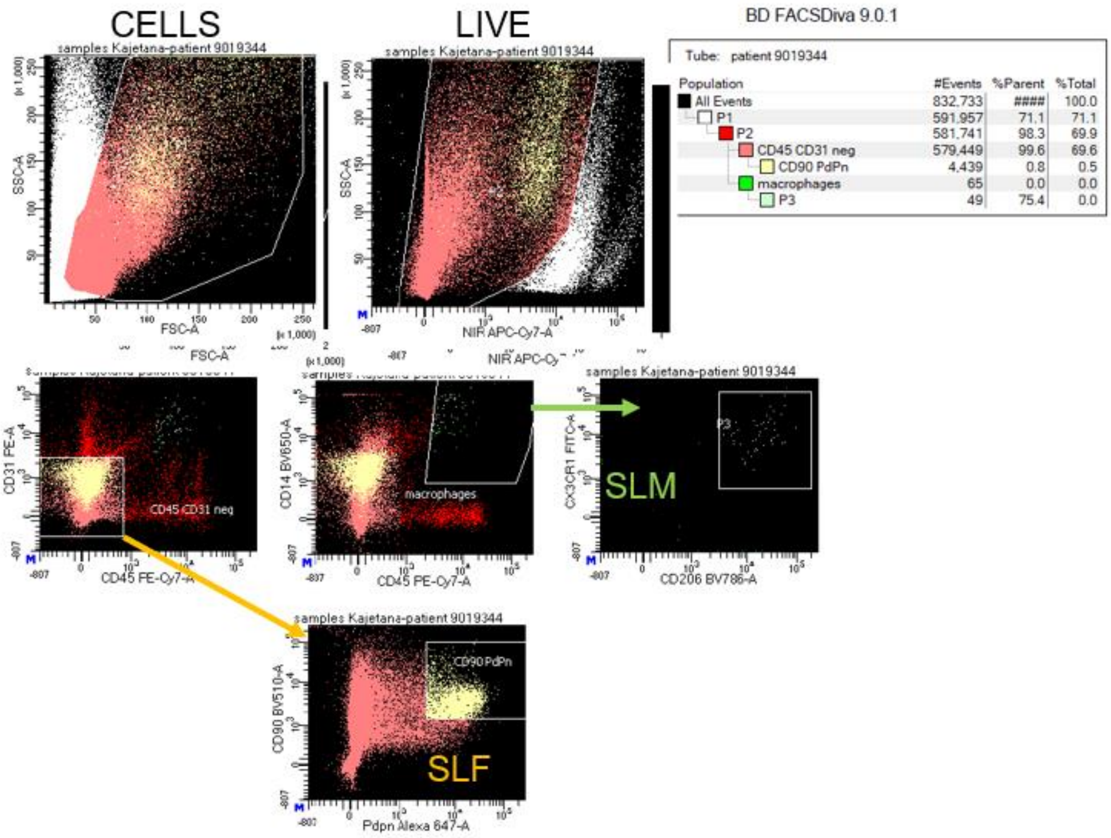

Figure S9 Synovial like Fibroblasts Sorting strategy

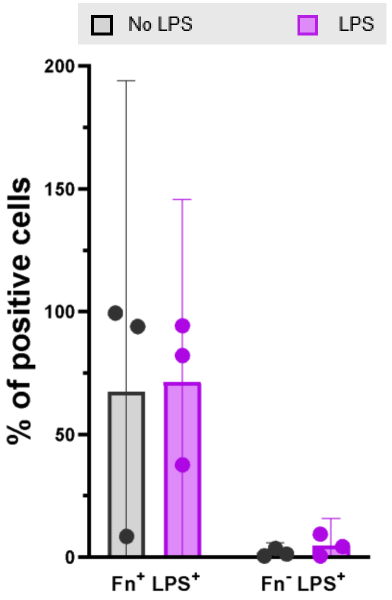

Figure S10 Quantification of Fibronectin and LPS single and double positive cells in the rat synovium

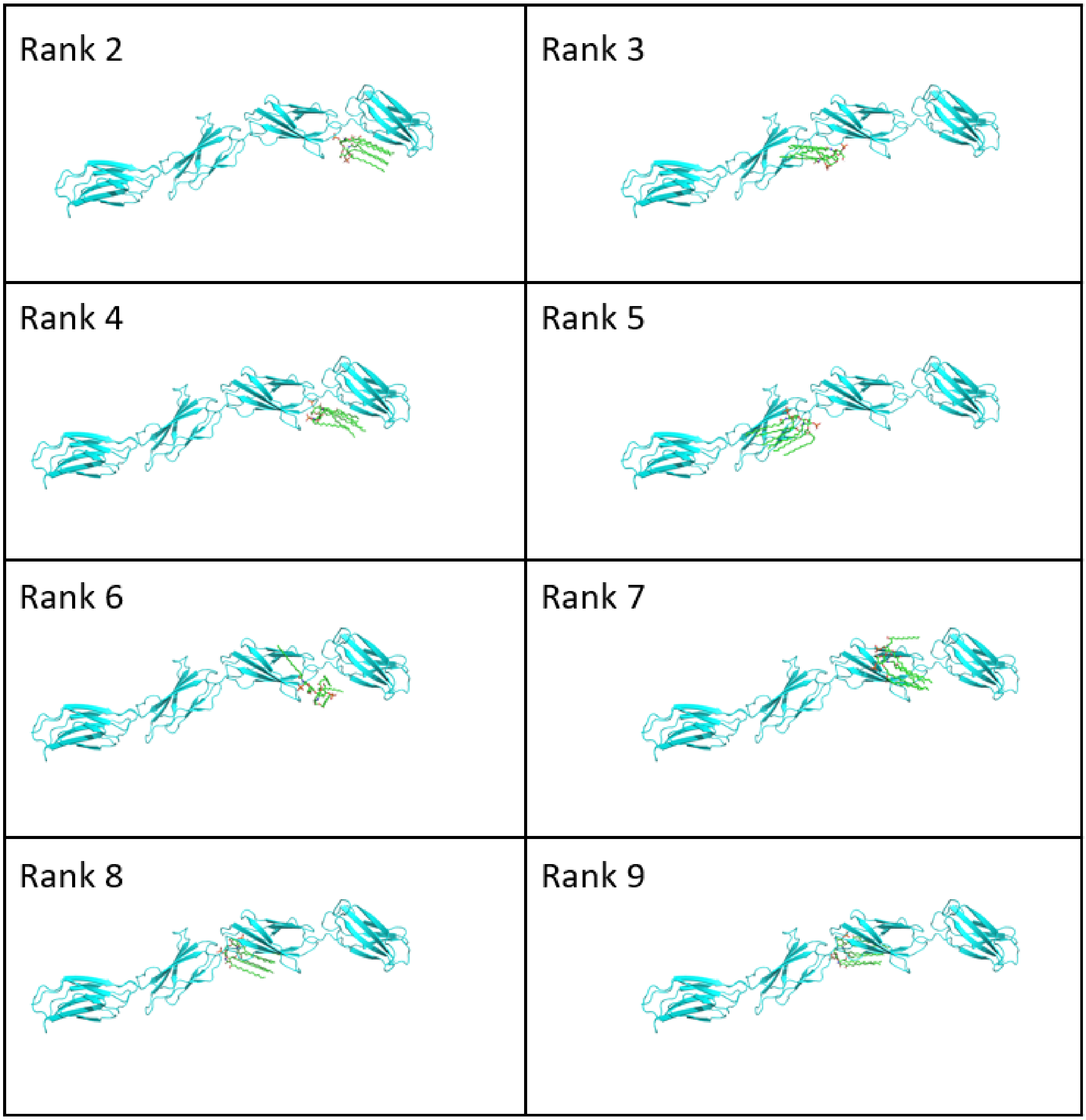

Figure S11 *In silico* prediction of binding of LPS and Fn domain III ranks 2-9
